# Association between homocysteine and white matter hyperintensities in rural‐dwelling Chinese people with asymptomatic intracranial arterial stenosis: A population‐based study

**DOI:** 10.1002/brb3.2205

**Published:** 2021-05-24

**Authors:** Xiang Wang, Hao Yin, Xiaokang Ji, Shaowei Sang, Sai Shao, Guangbin Wang, Ming Lv, Fuzhong Xue, Yifeng Du, Qinjian Sun

**Affiliations:** ^1^ Department of Neurology Shandong Provincial Hospital Affiliated to Shandong First Medical University Jinan China; ^2^ Department of Neurology Jining No.1 Peopleʹs Hospital Jining China; ^3^ Department of Biostatistics School of Public Health Shandong University Jinan China; ^4^ Department of Clinical Epidemiology Qilu Hospital Cheeloo College of Medicine Shandong University Jinan China; ^5^ Department of Radiology Shandong Medical Imaging Research Institute Cheeloo College of Medicine Shandong University Jinan China

**Keywords:** arteriosclerosis, asymptomatic intracranial arterial stenosis, homocysteine, population‐based study, white matter hyperintensities

## Abstract

**Purpose:**

Although homocysteine (Hcy) has been proven to be associated with the incidence of white matter hyperintensities (WMH) in patients with stroke, this association remains unclear in participants with asymptomatic intracranial arterial stenosis (aICAS). This study aimed to investigate the association of Hcy with WMH in participants with aICAS.

**Materials and methods:**

This was a cross‐sectional study based on the Kongcun Town Study. Participants diagnosed with aICAS by magnetic resonance angiography in the Kongcun Town Study were enrolled in this study. Data on demographics, lifestyle, medical histories, and Hcy levels were collected via interviews, clinical examinations, and laboratory tests. The volume of WMH was calculated using the lesion segmentation tool system for the Statistical Parametric Mapping package based on magnetic resonance imaging. The association between Hcy and WMH volume was analyzed using linear and logistic regression analyses.

**Results:**

A total of 137 aICAS participants were enrolled in the present study. Hcy was associated with the incidence of severe WMH (4th quartile, ≥4.20 ml) after adjustment for certain covariates [Hcy as a continuous variable, odds ratio (95% confidence interval) (OR (95% CI)): 1.09 (1.00, 1.19), *p* = .047; as a categorical variable (Hcy ≥15 μmol/L), OR (95% CI): 3.74 (1.37, 10.19), *p* = .010)]. After stratification according to the degree of aICAS, this relationship remained significant only in the moderate‐to‐severe stenosis group (stenosis ≥50%). (Hcy as continuous variable, OR (95% CI): 1.14 (1.02, 1.27), *p* = .025; as a categorical variable (Hcy ≥15 μmol/L), OR (95% CI): 5.59 (1.40, 15.25), *p* = .015).

**Conclusion:**

Serum Hcy concentration may be positively associated with the volume of WMH in rural‐dwelling Chinese people with moderate‐to‐severe (stenosis ≥50%) aICAS.

## Graphical abstract

This study investigated the association between homocysteine (Hcy) and white matter hyperintensities (WMH) in rural‐dwelling Chinese people with asymptomatic intracranial arterial stenosis (aICAS). The result suggested that Serum Hcy concentration may be associated with increased WMH volume in rural‐dwelling Chinese people with moderate‐to‐severe (stenosis≥50%) aICAS.

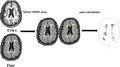

## 1 INTRODUCTION

White matter hyperintensities (WMH) are a type of cerebral small vessel disease characterized by hyperintensities on T2‐weighted magnetic resonance imaging (MRI) scans; these hyperintensities are predominantly located in the bilateral and symmetrical white matter of the brain (Garde et al., 2000; Pantoni, 2010). Pathological changes associated with WMH are mainly axonal degeneration, demyelination, oligodendrocyte apoptosis (Wardlaw et al., 2019). It has been demonstrated that WMH is associated with an increased risk of ischemic stroke (Conijn et al., 2011; Selvarajah et al., 2009), cognitive decline (Silbert et al., 2008), and dementia (Kester et al., 2014). With the emerging trend of social aging, the incidence of WMH‐related diseases may increase and these may pose a serious threat to human health. The pathogenesis of WMH remains unclear; however, chronic cerebral ischemia, hyperperfusion injury, endothelial dysfunction, direct damage to the blood‐brain barrier, and inflammatory and genetic factors are hypothesized to be potential causes of WMH (Joutel & Chabriat, 2017).

Homocysteine (Hcy) is a sulfur‐containing amino acid formed during methionine metabolism. When the metabolic pathway for methionine is impaired, the plasma concentration of Hcy increases, which may induce a series of vascular pathological changes. In recent years, studies have reported that increased Hcy levels were associated with a high WMH volume in both the general population and patients with stroke, with the pathophysiological mechanism for this being related to endothelial dysfunction leading to microvascular disease (Naveed & Bokhari, 2015; Vermeer et al., 2002; Wright et al., 2005). However, data on the relationship between Hcy and WMH are still lacking in patients with asymptomatic intracranial arterial stenosis (aICAS). As an intermediate state, research about the relationship between Hcy and WMH in this period will help us obtain more information that cannot be obtained from studies on the general population and clinical stroke patients. This may be very helpful for a comprehensive understanding of the relationship between Hcy and WMH. It is assumed that in participants with aICAS, high levels of Hcy may be associated with an increase in WMH volume. Moreover, considering that intracranial stenosis may play an important role in the pathophysiology of WMH, and patients with severe ICAS are more likely to have more serious WMH (Park et al., 2015), it is hypothesized that the effects of Hcy on WMH may be more pronounced in patients with severe stenosis.

Therefore, in the present study, we tested this hypothesis by investigating the association between Hcy and WMH in rural‐dwelling Chinese people with aICAS.

## 2 MATERIALS AND METHODS

### 2.1 Study population

The current participants originated from the Kongcun Town Study, an ongoing longitudinal study mainly aimed to investigate: (1) the occurrence and distribution of aICAS; (2) major cardiovascular risk factors or biomarkers related to the development and prognosis of aICAS. The detailed study design of Kongcun Town Study has been described previously (Sun et al., 2020; Wang, Wang, et al., 2020; Wang, Zhao, et al., 2020; Xue et al., 2020). In brief, from October 2017 to May 2018, all participants aged ≥40 years who were free of a history of clinical stroke or transient ischemic attack (TIA) were enrolled. Participants with severe cardiopulmonary insufficiency, vision, or hearing impairments were excluded. The baseline investigation of the Kongcun study included two phases: The phase 1 assessment aimed to collect data (demographic features, health history, lifestyle factors, etc.) and identify people at high risk of aICAS using transcranial Doppler (TCD) examination. In phase 2, those who screened positive for aICAS further underwent structural brain MRI and magnetic resonance angiography (MRA). After a baseline line investigation, 204 participants were positive for aICAS by TCD; of them, 169 participants completed MRI/MRA. Among these 169 participants, 15 subjects were excluded because of previous silent cerebral infarction detected by MRI, and finally, 154 were diagnosed with aICAS. All the 154 participants were included in the present study. Of the 154 participants, 10 participants were excluded because of missing Hcy data, and another seven participants were excluded due to unqualified MR images. Consequently, all analyses were based on 137 participants with complete baseline data on Hcy and MR images.

### 2.2 Ethics approval and consent to participate

The study protocol was reviewed and approved by the ethical standards committees on human experimentation at Shandong Provincial Hospital Affiliated to Shandong First Medical University. Written informed consent was obtained from all the participants.

### 2.3 Assessments of aICAS

Asymptomatic intracranial arterial stenosis was defined as ICAS without a history of stroke or TIA and previous silent cerebral infarction detected by MRI in the present study. A self‐report of a physician diagnosis and/or clinical examinations showing typical symptoms were considered as a history of stroke or TIA, which was determined during the interviews and clinical examination. Participants were examined using a portable TCD machine (VIASYS Companion III, Nicolet, Washington, United States) by two experienced physicians. Those who screened positive according to the criteria for intracranial stenosis by TCD further underwent MRA (Feldmann et al., 2007). The extent of stenosis in the five evaluated arteries (bilateral middle cerebral arteries, bilateral intracranial segment of internal carotid arteries, and basilar artery) was classified into five grades based on the signal strength of MRA detection: normal, mild (signal reduction <50%), moderate (signal reduction ≥50% and <70%), severe (signal reduction ≥70%), or occlusion (focal signal loss with the presence of distal signal). Detailed information on the assessments of aICAS can be found in previous studies (Wang, Wang, et al., 2020; Wang, Zhao, et al., 2020).

### 2.4 WMH volume measurement

**FIGURE 1:**
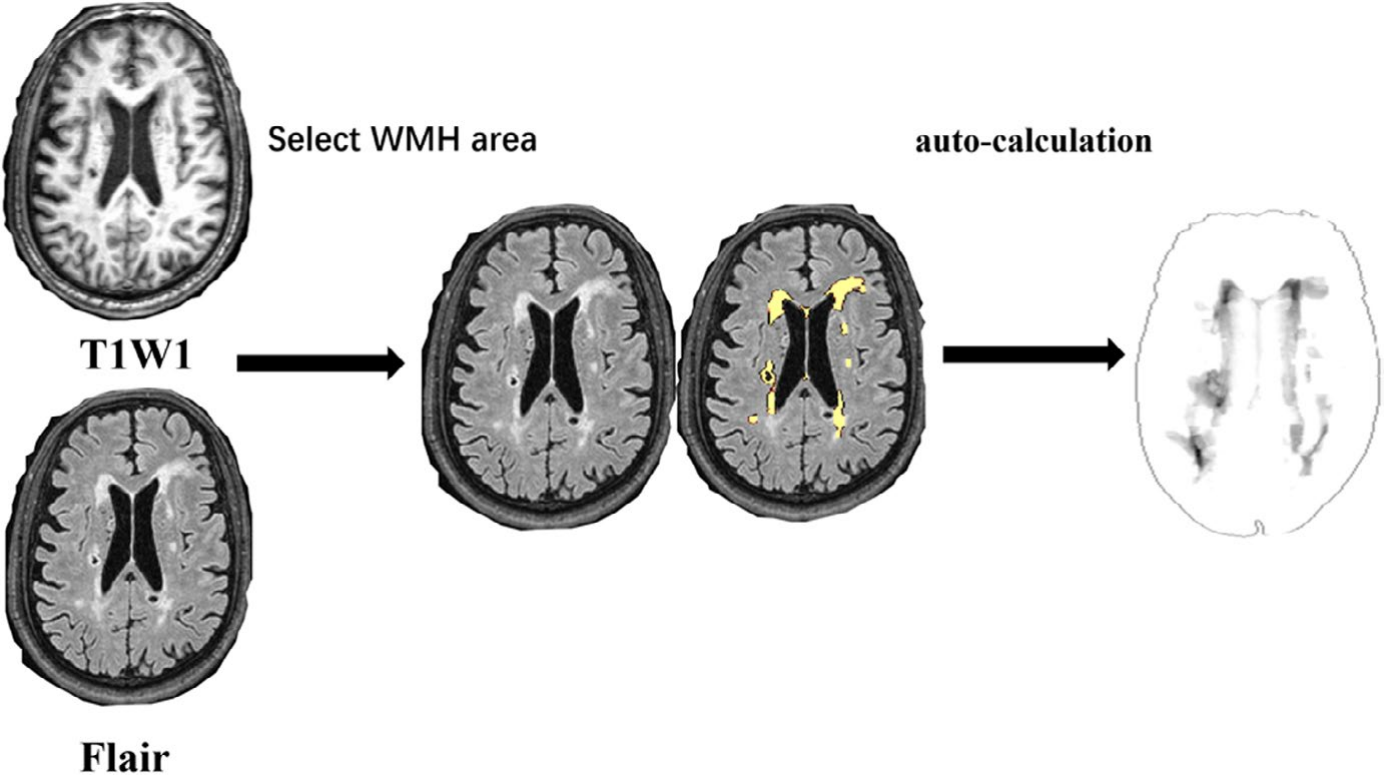
​Flowchart of the measurement of white matter hyperintensities volume using lesion segmentation tool auto‐calculation system

Participants who screened positive by TCD received a brain MRI assessment using a 3‐T magnetic resonance scanner (Philips, Achieva, Netherlands), including T1‐weighted images, T2‐weighted images, proton density images, T2 fluid‐attenuated inversion recovery images, and diffusion‐weighted imaging, as described previously (Sun et al., 2020). WMH was calculated by two trained investigators and a neuroradiologist blinded to clinical information using the lesion segmentation tool (LST) for the Statistical Parametric Mapping package (SPM) (Schmidt et al., 2019). WMH was defined as hyperintensities on proton density and T2‐weighted images, with no prominent hypointensity on T1‐weighted images. The volume of WMH was divided into four categories; patients with WMH volumes in the highest (4th) quartile were classified into the severe WMH group (≥4.20 ml). Figure 1 shows a flowchart of the WMH volume using LST auto‐calculation.

### 2.5 Hcy and covariates

Trained investigators conducted face‐to‐face questionnaires, physical examinations, and anthropometric measurements at the participants’ homes. Peripheral venous blood samples were collected in the morning after overnight fasting at a local clinic. Blood tests for fasting blood glucose, serum Hcy, and lipid profiles and biochemical tests including low‐density lipoprotein‐cholesterol, high‐density lipoprotein‐cholesterol, triglyceride, total cholesterol, and high‐sensitivity C‐reactive protein were conducted at the certified clinical laboratory of the Shandong Provincial Hospital Affiliated to Shandong First Medical University. We measured serum Hcy levels using a method for commercial use [circulating enzyme/lactate dehydrogenase method, Zhejiang Dongou Diagnostic Products Co, Ltd, Zhejiang, China; CV measurements ≤10%, total Hcy concentration (μmol/L) = (∆A/min sample ‐∆A/min blank)/(∆A/min calibration ‐∆A/min blank)]. The principle of this method was as follows: Step one: oxidized Hcy was converted into free Hcy. Consequently, it reacted with serine and turned into L‐cystathionine under the catalysis of cystathione β‐synthase; Step two: L‐cystathionine was further transformed into Hcy, pyruvic acid, and NH_3_ under the catalysis of cystathione β‐catabolase; Step three: pyruvate produced by the above cyclic reaction was detected by lactic dehydrogenase and oxidized nicotinamide adenine dinucleotide (NADH), and the rate of conversion of NADH to nicotinamide adenine dinucleotide was directly proportional to the Hcy content. High levels of Hcy (hHcy) were defined as serum Hcy ≥15 μmol/L, which is a commonly used standard (Sachdev, 2004; Wright et al., 2005). The height and weight of participants were measured without shoes, and body mass index (BMI) was calculated (kg/m^2^). Blood pressure (mmHg) was measured twice with a sphygmomanometer after a 5‐min rest each time. Hypertension was defined as blood pressure ≥140/90 mmHg or the use of antihypertensive drugs. Diabetes mellitus was defined as a history of diabetes, a glucose level of ≥7.0 mmol/L, or self‐reported use of oral antidiabetic drugs or insulin. Hypercholesterolemia was defined as total cholesterol ≥6.20 mmol/l or an ongoing prescription of hypolipidemic drugs. Smoking was assessed by enquiring about the amount of packs smoked per year, and alcohol intake was categorized as never, former, or current. Carotid ultrasound examination was performed by two experienced physicians in a local clinic to measure extracranial arterial stenosis (ECAS) with a 7 MHz linear transducer (Siemens ACUSON P500) according to the peak systolic blood flow velocity (Grant et al., 2003). A diagnosis of ECAS was established when one or more of the following arteries were observed to be stenotic to any degree by ultrasound: the common carotid artery, ICA, and external carotid artery.

### 2.6 Data analysis

Data are shown as mean (Standard Deviation, *SD*) or median (interquartile range) for continuous variables and frequencies (%) for categorical variables. We compared baseline demographics, vascular risk factors, and serum Hcy levels between patients in the 4th quartile, who had the highest WMH volume (≥4.20 ml) and those in the 1st–3rd quartiles, who had a lower volume of WMH (<4.20 ml), using the *t* test (for means), Wilcoxon's rank‐sum test (for medians), and Chi‐square test (for percentage). The association between Hcy (as continuous and binary variables) and WMH volume was examined by a generalized inear regression analysis (if WMH volume was considered a continuous variable, simple linear regression was performed; if it was considered a categorical variable (for severe and mild to moderate groups), logistic regression was performed), followed by a multiple linear regression analysis. Covariates with *p* <.10 in the univariate analysis were further incorporated into the multivariate regression analysis for adjustment. The association between Hcy and WMH volume was further examined after stratifying the stenosis in the intracranial arteries into mild (MRA signal reduction <50%) and moderate‐to‐severe groups (MRA signal reduction ≥50%). We used the statistical packages R (http://www.r‐project.org; version 3.4.3) and EmpowerStats (www.empowerstats. Com; X&Y Solutions Inc.) for all the analyses. Results with *p‐*values <.05 were considered statistically significant (2‐sided tests).

## 3 RESULTS

The mean age of the 137 participants was 59.90 [*SD*, 10.14] years; 81 (59.12%) of them were male. Forty‐eight (35.04%) participants were current smokers, and 43 (31.39%) were alcohol drinkers. A total of 108 patients (78.83%) had hypertension, 29 (21.17%) had hypercholesterolemia, and 37 (27.01%) had diabetes. Fourteen (10.22%) participants had ECAS, and 94 (68.61%) had moderate‐to‐severe aICAS. Details of the clinical and demographic features of the patients are presented in Table 1.

**TABLE 1:** Characteristics of participants according to severity of WMH

	All (*n* = 137)	WMH(ml)		*p*‐values
		1st −3rd quartile (*n* = 101)	4th quartile (*n* = 36)	
Age (years), mean (*SD*)	59.90 (10.14)	56.98(8.98)	68.08(8.69)	<.001
Sex (man), *n* (%)	81 (59.12)	66 (65.35)	15 (41.67)	.013
Smoking, *n* (%)	48 (35.04)	28 (27.72)	20 (55.56)	.003
Drinking, *n* (%)	43 (31.39)	26 (25.74)	17 (47.22)	.017
Hypertension, *n* (%)	108 (78.83)	76 (75.25)	32 (88.89)	.085
Diabetes, *n* (%)	37 (27.01)	27 (26.73)	10 (27.78)	.903
Hypercholesterolemia, *n* (%)	29 (21.17)	25 (24.75)	4 (11.11)	.085
Body mass index (kg/m^2^), mean (*SD*)	25.58 (3.15)	25.72(2.93)	25.19(3.73)	.385
Systolic pressure (mmhg), mean (*SD*)	157.46 (24.18)	153.68 (22.43)	167.75 (26.07)	.003
Diastolic pressure (mmhg),mean (*SD*)	91.95(13.36)	91.87(13.49)	92.17(13.18)	.909
FBG (mmol/l), mean (*SD*)	6.61 (1.94)	6.61(2.04)	6.63(1.68)	.957
Total cholesterol (mmol/l), mean (*SD*)	5.42 (0.95)	5.49 (0.96)	5.23(0.91)	.164
HDL‐c (mmol/l), mean (*SD*)	1.50 (0.31)	1.48 (0.31)	1.54 (0.32)	.331
LDL‐c (mmol/l), mean (*SD*)	3.15 (0.71)	3.20 (0.69)	3.01 (0.75)	.162
Triglycerides (mmol/l), mean (*SD*)	1.58 (1.08)	1.66 (1.15)	1.36 (0.82)	.147
HR‐CRP (mg/L), mean (*SD*)	1.75 (2.45)	1.54 (1.65)	2.33 (3.89)	.097
Hcy (μmol/l), mean (*SD*)	14.95 (6.54)	13.78 (5.32)	18.24 (8.38)	<.001
hHcy, *n* (%)	47 (34.31)	25 (24.75)	22 (61.11)	<.001
Extracranial arterial stenosis, *n* (%)	14 (10.22)	8 (7.92)	6 (16.67)	.137
Intracranial arterial stenosis (moderate‐to‐severe), *n* (%)	94 (68.61)	69 (68.32)	25 (69.44)	.900

Note

*p*‐values are for the test of differences between 1st –3rd and 4th quartile of WMH volume.

Abbreviations: FBG, Fasting blood glucose; Hcy, total homocysteine; HDL‐c, high‐density lipoprotein cholesterol; hHcy, high level of homocysteine (≥15mmol/l); HR‐CRP, high‐sensitivity c‐reactive protein; LDL‐c, low‐density lipoprotein cholesterol; WMH, white matter hyperintensity; the 4th quartile : volume of WMH ≥4.20 ml); 1st–3rd quartile, volume of WMH <4.20 ml.

Among these 137 patients, 36 (26.28%) were in the 4th quartile group of WMH volume, and 15 of these (41.67%) were male. Compared to patients in the 1st –3rd quartile group, patients in the 4th quartile group were older (*p* < .001), had a higher systolic pressure (*p* = .003) and were more likely to smoke (*p* = .003) and drink (*p* = .017). The mean serum Hcy level was also significantly higher in the 4th quartile group than in the 1st –3rd quartile group (*p* <.001). The proportion of participants with hHcy was also significantly higher in the 4th quartile than in the 1st –3rd quartile group (*p* < .001). There were no significant differences in other variables between the two groups (Table 1).

The multiple regression analysis showed that when WMH was analyzed as a continuous variable, Hcy was not significantly associated with WMH after adjusting for covariates. However, as a categorical variable (for 4th and 1st–3rd quartile groups), the serum Hcy level was independently associated with a high volume of WMH after adjustment for certain covariates (Hcy as continuous variable: OR (95% CI): 1.09 (1.00, 1.19), *p* = .047; Hcy as categorical variable: (Hcy ≥15 μmol/L), OR (95% CI): 3.74 (1.37, 10.19), *p* = .010) (Table 2). When further stratified according to the degree of intracranial arterial stenosis, the association between Hcy level and WMH volume was significant only in the moderate‐to‐severe stenosis group (Hcy as continuous variable: OR (95% CI): 1.14 (1.02, 1.27), *p* = .025; Hcy as categorical variable (Hcy ≥15 μmol/L): OR (95% CI): 5.59 (1.40, 15.25), *p* = .015) (Table 3).

**TABLE 2:** Association between Hcy and WMH by generalized regression analysis

	Nonadjusted	*p*	Adjusted	*p*
	β/OR (95%CI)		β/OR (95%CI)	
WMH (ml)				
Hcy (μmol/L)	0.20 (0.06, 0.34)	.006	0.07 (−0.07, 0.21)	.344
hHcy	3.30 (1.39, 5.22)	.001	1.82 (−0.03, 3.67)	.056
Severe WMH				
Hcy (μmol/L)	1.11 (1.04, 1.18)	.002	1.09 (1.00, 1.19)	.047
hHcy	4.78 (2.13, 10.72)	.001	3.74 (1.37, 10.19)	.010

Note

Nonadjusted model adjust for: None.

Adjust model adjust for: age; sex; smoking; drinking; hypertension; hypercholesterolemia; high‐sensitivity c‐reactive protein; WMH: white matter hyperintensity; Severe WMH (binary variable): highest (4th) quartile of WMH (volume≥4.20 ml); Hcy: homocysteine; hHcy (binary variable): high level of homocysteine (≥15 μmol/l).

**TABLE 3:** Association between Hcy and WMH according to different degree of intracranial arterial stenosis

	Mild aICAS (stenosis <50%)	*p*	Moderate‐to‐severe aICAS (stenosis≥50%)	*p*
	β/OR (95%CI)		β/OR (95%CI)	
WMH (ml)				
Nonadjusted				
Hcy (μmol/L)	0.21 (−0.13, 0.55)	.2377	0.20 (0.03, 0.36)	.022
hHcy	3.48 (0.65, 6.30)	.0203	3.17 (0.69, 5.65)	.014
Adjusted				
Hcy (μmol/L)	0.04 (0.32, 0.39)	.8467	0.08 (−0.09, 0.24)	.362
hHcy	2.40 (−0.33, 5.13)	.0944	1.63 (−0.82, 4.08)	.197
Severe WMH Nonadjusted				
Hcy (μmol/L)	1.07 (0.90, 1.27)	.4500	1.12 (1.04, 1.20)	.003
hHcy	3.61 (0.82, 15.90)	.0895	5.59 (2.07, 15.09)	.001
Adjusted				
Hcy (μmol/L)	0.98 (0.72, 1.33)	.8972	1.14 (1.02, 1.27)	.025
hHcy	3.07 (0.37, 15.59)	.3000	5.59 (1.40, 15.25)	.015

Note

Nonadjusted model adjust for: None.

Adjust model adjust for: age; sex; smoking; drinking; hypertension; hypercholesterolemia; high‐sensitivity c‐reactive protein.

Abbreviations: aICAS, asymptomatic intracranial arterial stenosis; Hcy, homocysteine; hHcy (binary variable), high level of homocysteine (≥15 μmol/L); WMH, white matter hyperintensity; Severe WMH (binary variable)**:** highest (4th) quartile of WMH (volume≥4.20 ml).

## 4 DISCUSSION

The results of this study revealed that serum Hcy levels may be independently associated with the severity of WMH in rural Chinese people with moderate‐to‐severe ICAS (MRA signal reduction ≥50%).

Previous studies on the impact of Hcy on WMH have been carried out in the general population, with inconsistent results. The Northern Manhattan Study found that higher levels of Hcy were associated with WMH after adjusting for sociodemographics and vascular risk factors in a community‐based sample (Wright et al., 2005). Several other studies involving community‐based participants also reported a similar association between Hcy and WMH (Raz et al., 2012; Sachdev et al., 2004; Vermeer et al., 2002). However, two studies failed to find any association between WMH and Hcy in general participants, one study aimed to evaluate the relationship between Hcy levels and findings on brain MRI and found that higher plasma Hcy levels are associated with a smaller brain volume but not with WMH (Seshadri et al., 2008). In another study, after adjusting for age and sex, Hcy levels were not found to be associated with WMH (Longstreth et al., 2004). Different study populations, age ranges, sample sizes, and ethnicities may have led to the differing results in these studies.

Unlike the results drawn for general participants, in cross‐sectional studies involving patients with stroke (Tseng et al., 2009), Parkinson's disease (Shen et al., 2019), or rheumatoid arthritis (Anan et al., 2009), researchers have reached a relatively consistent conclusion that Hcy is a risk factor for cerebral white matter lesions. Prospective studies have also obtained similar results. For example, in a longitudinal population‐based study involving participants aged 60 years or older who were free of dementia, the total Hcy concentration was confirmed to be related to the progression of WMH (Hooshmand et al., 2016). Our study is consistent with these studies, and to the best of our knowledge, this is the first study to focus on the association between Hcy and WMH in participants with aICAS. A noteworthy finding of our study was that the relationship between Hcy and WMH was only significant in the moderate‐to‐severe stenosis (≥50%) aICAS group after adjusting for confounders. This finding suggests that, to a certain extent, stenotic intracranial arteries may be involved in the pathophysiology of WMH caused by Hcy, even in the absence of clinical stroke. Compared with general participants or those with mild stenotic aICAS, participants with greater than 50% stenosis of an intracranial artery may have more serious vascular pathological changes, including large conduit vessels and microcirculation (Ritz et al., 2014), which may lead to the brain tissue being more vulnerable to abnormal Hcy levels. The mechanisms by which Hcy causes the incidence of WMH are: 1) inhibition of endothelial nitric oxide via the production of reactive oxygen species or the accumulation of asymmetric dimethylarginine, leading to functional suppression of the blood‐brain barrier (BBB) (Kamath et al., 2006; Woo et al., 1997), infiltration of toxic materials into neural tissues, and blockage of interstitial fluid clearance (Doubal et al., 2010; Hainsworth & Fisher, 2017; Hassan et al., 2003); 2) activation of the coagulating pathway by increasing the expression of prothrombotic factors, such as β‐thromboglobulin, tissue plasminogen, and factor VIIc (Nishinaga et al., 1993; Schreiner et al., 2002); and 3) promoting the growth of vascular smooth muscle cells (Chen et al., 2000).

This study has the following strengths: First, to the best of our knowledge, this is the first study to explore the association between Hcy and WMH in rural‐dwelling Chinese people with aICAS. As a high‐risk group for stroke, research on these participants helps us provide more data on the relationship between Hcy and WMH, and further explore the possible mechanisms for Hcy causing WMH. Second, the use of automated, quantitative measurements of WMH avoided the subjective factors of previously used semiquantitative methods and may have produced more reliable results. For example, in another study using automatic quantitative segmentation methods to measure the volume of WMH, the upper quartile range of WMH volume was greater than or equal to 5.93 ml (4th Q ≥ 5.93 ml), which is basically consistent with the ranges of the present study (Wong et al., 2006). Indeed, the lesion prediction algorithm implemented in SPM's LST toolbox has been shown to produce consistently good performance and in many cases is considered a robust gold standard for lesion segmentation (Guerrero et al., 2018). However, there are several limitations to be addressed. First, when WMH was used as a continuous variable, the relationship between Hcy and WMH was not significant, and when Hcy was analyzed as a binary variable (Hcy ≥15 μmol/L or not), the 95% CI of the OR was relatively wider than when Hcy was analyzed as a continuous variable. However, point estimation of the OR indicates a significant association between Hcy and WMH, we believe that the small sample size in the present study may be one of the main reasons for these results. As a single‐center, exploratory study with a relatively small sample size, the present study could not reach a definite conclusion on the association between Hcy and WMH. Multi‐center prospective studies with larger sample sizes are required to confirm whether elevated Hcy causes progression of white matter damage, and whether there is a causal pathway between elevated serum Hcy levels and outcomes such as stroke and cognitive decline. Second, it has been recognized that folate and vitamin B12 levels have an effect on the level of serum Hcy (Jacques et al., 2001); however, data on serum folate and vitamin B12 levels are lacking in the present study, and the effect of these vitamins on the variation of Hcy level could not be examined. Third, it has also been reported that the association between Hcy and WMH may differ depending on the location of the brain being examined (Gao et al., 2015). Our study did not compare the incidence of WMH in different locations (periventricular versus. deep WMH); therefore, the association between WMH and Hcy in different parts of the brain could not be ascertained.

## 5 CONCLUSION

In conclusion, the present study showed that Hcy is a risk factor for white matter lesions in patients with moderate‐to‐severe aICAS (≥50%) in rural Chinese dwellings. Future prospective studies with a larger sample size are warranted to confirm our findings. At the same time, further studies including healthy individuals without ICAS will help to clarify whether aICAS is involved in the pathophysiological pathway of WMH caused by Hcy.

## CONFLICT OF INTERESTS

The authors declare that they have no competing interests.

## AUTHORS CONTRIBUTIONS

QJS, YFD, and FZX conceived and designed the research. WX, HY, XKJ, SWS, SS, PY, SL, and YX acquired the data. WX, SS, PY, SL, YX, and GBW analyzed and interpreted the data. WX and HY draft the manuscript. ML, FZX, YFD, and QJS made critical revisions to the manuscript. All authors approved the final manuscript.

## ETHICAL APPROVAL

This study was approved by the ethical standards committee on human experimentation at Shandong Provincial Hospital Affiliated to Shandong First Medical University.

## CONSENT TO PUBLISH

Not applicable.

### PEER REVIEW

The peer review history for this article is available at https://publons.com/publon/10.1002/brb3.2205.

## Data Availability

The data that support the findings of this study are available from the corresponding author upon reasonable request.
